# Microvascular angiotensin II type 2 receptor function is enhanced in young females and declines in a model of murine aging

**DOI:** 10.20517/jca.2024.09

**Published:** 2024-08-14

**Authors:** Casey G. Turner, Karla de Oliveira, Qing Lu, Ayan R. Patel, Lakshmi Pulakat, Iris Z. Jaffe, Jennifer J. DuPont

**Affiliations:** 1 Molecular Cardiology Research Institute, Tufts Medical Center, Boston, MA 02111, USA.; 2 Division of Cardiology, Department of Medicine, Tufts Medical Center, Boston, MA 02111, USA.

**Keywords:** Microvascular function, aging, angiotensin II type 2 receptor, sex differences, skin blood flow, cutaneous

## Abstract

**Introduction:**

Angiotensin II (AngII) affects cardiovascular health, mediating impacts through AngII type 1 (AT1R) and type 2 (AT2R) receptors. The present study investigated sex and aging-related differences in microvascular AngII receptor function in mice and humans.

**Methods:**

Mesenteric resistance arteries (MRA) were isolated from 3-, 12-, and 18-month-old female and male C57/Bl6 mice. Wire myography was used to measure vasoconstriction to AngII and vasodilation to an AT2R agonist (compound 21, C21). Seven healthy adults (3 premenopausal women and 4 age-matched men) were recruited to participate in a study measuring cutaneous microvascular vasoconstriction to AngII in the presence and absence of 10 μM PD123319, an AT2R antagonist.

**Results:**

In murine MRA, AngII-induced constriction increases by 18 months in females and by 12 months in males. AT2R-mediated vasodilation was reduced with age in females only, which corresponds with a female-specific decrease in mesenteric AT2R mRNA expression. AT2R inhibition enhances AngII-induced constriction in young female, but not male, mice. Clinical data support that premenopausal women have attenuated AngII constriction *vs*. men, which is abrogated by AT2R inhibition. AT2R expression is greater in primary aortic smooth muscle cells, but not endothelial cells, from young women compared with men.

**Conclusions:**

These data demonstrate enhanced microvascular AT2R function in young female mice and young women. There is a female-specific loss of AT2R function with age in mice, concomitant with declining AT2R expression. These findings implicate AT2R as a sex-specific target for microvascular dysfunction and aging-associated cardiovascular disease.

## Graphical Abstract

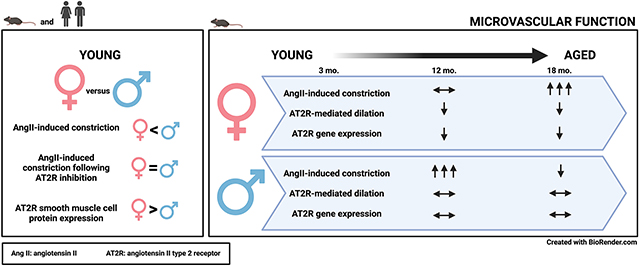

## BACKGROUND

There are significant sex differences in the prevalence and rate of progression of cardiovascular diseases across the lifespan, including in myocardial infarction, stroke, heart failure, and hypertension^[1,2]^, yet the mechanisms for these sexual dimorphisms remain largely unknown. The average age of hypertension onset and cardiovascular disease development is delayed in women compared with men, with prevalence increasing in women after menopause and ultimately exceeding that of men^[1]^. To date, sex-specific therapeutic targets for hypertension and cardiovascular disease have yet to be identified, which is a critical step toward improving cardiovascular outcomes and attenuating health disparities in women across the lifespan.

The renin-angiotensin-aldosterone system (RAAS) is a hormonal cascade that regulates blood pressure and plays an important role in cardiovascular health and disease^[3–5]^. Components of the RAAS are altered with aging in humans and rodents, including the levels of circulating hormones, expression of the enzymes that produce these hormones, and the receptors that mediate their impacts on cardiovascular tissues^[3–6]^. Angiotensin II (AngII) is a circulating peptide hormone and a main effector of the RAAS. There are two main receptor subtypes through which AngII exerts its effects: AngII type 1 receptors (AT1R) and type 2 receptors (AT2R)^[7]^. The AT1R exerts classical AngII effects in the cardiovascular system, including mediating potent vasoconstriction^[7]^. Conversely, the AT2R exerts opposing cardiovascular effects, specifically inducing vasodilation^[7–11]^, which modulates the vasoconstrictive and pro-hypertensive actions of Ang II.

The microvasculature is an important contributor to blood pressure regulation by controlling peripheral vascular resistance. As such, the balance between AT1R- and AT2R-mediated effects in the microvasculature contributes to the development of hypertension^[12]^ and other cardiovascular conditions^[9]^. Both AT1R and AT2R are expressed in endothelial cells and vascular smooth muscle cells in mesenteric resistance arteries (MRA) in rodents^[9,10]^. AT2R expression is greatest during development, and though it declines to lower absolute and relative (*vs*. AT1R) levels in adulthood^[9,12–15]^, the AT2R functionally contributes to blood pressure control, renal physiology, and microvascular function in adult rodent models^[10,12]^ and humans^[15–17]^. These findings, plus the recent development of AT2R agonists for human health^[18,19]^, heighten interest in the role of AT2R in the cardiovascular system.

Data from animal models and recent human studies suggest sex- and aging-related differences in AT2R function and expression in the cardiovascular system^[11,16,17,20–23]^. The gene that encodes for AT2R is located on the X chromosome^[24]^, and estrogen and testosterone modulate AT2R expression in the vasculature of rodents^[25,26]^. Sex hormone receptors also mediate AngII-induced hypertension and AngII downstream signaling^[9,20–22]^. In humans, young women have enhanced cutaneous microvascular vasodilation in response to AT2R activation^[17]^, and older adults have enhanced microvascular constriction in response to AngII than younger adults, a difference which can be abolished by AT2R inhibition^[16]^. Detailed investigation of the contribution of AT2R to microvascular function during aging has been limited and many preclinical studies have been performed only in male rodents; thus, there is a knowledge gap regarding potential sex differences in microvascular AngII receptor function in young and aged models.

Here, we investigate sex differences in AngII-induced microvascular constriction and AT2R-mediated dilation in an aging murine model and relate the murine model findings with clinical microvascular function studies in humans. We hypothesized that: (1) AngII-induced constriction would increase later in life in female compared with male mice; (2) AT2R function would be greater in female compared with male mice and lost with aging; and (3) AngII vasoconstriction in young female mice and women would be reduced compared to males and that this difference would be abolished with AT2R antagonism.

## MATERIALS & METHODS

### Animal studies

All mice were handled in accordance with US National Institutes of Health standards, and all procedures were approved by the Tufts University Institutional Animal Care and Use Committee (Protocol #: B2023–83). C57/Bl6J male and female mice were utilized for all animal studies. Mice were studied at 3 (young), 12 (middle-aged), and 18 (older-aged) months of age, as these ages are analogous with sexual dimorphisms in the timing of the development of vascular aging in humans^[6]^. These ages also model some aspects of the progression of sex hormone availability in aging women, where 3-month-old mice have achieved reproductive maturity, 12-month-old female mice have irregular estrous cycling and decreased fertility, and 18-month-old female mice are post-reproductive^[6,27]^.

### Mesenteric vessel wire myograph studies

Rings from second- and third-order mesenteric resistance arteries (MRA) were mounted in a myograph (Danish Myo Technologies) for isometric tension recordings using PowerLab software (AD Instruments). A total of four rings per mouse were used for each wire myograph study. Rings were placed under a resting tension of 2 milliNewtons (mN) in tissue baths containing warmed (37 °C), aerated (95% O_2_, 5% CO_2_) standard physiological saline solution (PSS) (in mM: 130 NaCl, 4.7 KCl, 1.17 MgSO_4_, 0.03 EDTA, 1.6 CaCl_2_, 14.9 NaHCO_3_, 1.18 KH_2_PO_4_ and 5.5 glucose). Administration of 10 μM phenylephrine (PE) was used to test arterial viability, and the presence of intact endothelium was verified by acetylcholine (Ach, 1 μM)-induced relaxation of a half-maximal PE-induced contraction. Only vessels that met these criteria (intact SMC and endothelium) were included in the results. For vasoconstriction studies, four concentrations of AngII were administered (1 × 10^−9^, 3 × 10^−9^, 1 × 10^−8^, 1 × 10^−7^ M) to four separate mesenteric vessel rings (one concentration per ring due to AT1R internalization), and raw force was measured in mN. For vasodilation studies, vessels were pre-treated with 10 μm losartan for 30 min to block AT1R. Vessels were then preconstricted with PE and a dose-response of AT2R agonist compound-21 (C21, a gift from Vicore Pharma; 1 × 10^−12^-1 × 10^−7^ M) was administered. Finally, to interrogate the role of AT2R in AngII-induced vasoconstriction, vessels were pre-treated with vehicle or 10 μM PD123319, an AT2R antagonist, and raw force was measured in mN in response to one dose of 10^−7^ M AngII.

### Quantitative RT-PCR

Total RNA was extracted from mouse MRA, reverse transcribed, and quantitative RT-PCR was performed with gene-specific primers, as previously described^[28,29]^. Each *N* represents MRA from two mice pooled together to maximize the total RNA isolated. *C*_*t*_ values were normalized to β2-microglobulin (*B2m*). Primer sequences are specified in Supplementary Table 1.

### Human microvascular function studies

In vivo studies in humans were approved by the Tufts Health Sciences Institutional Review Board and conformed to the guidelines set forth by the Declaration of Helsinki. All participants gave verbal and written informed consent before participation. Seven healthy individuals (3 premenopausal women and 4 age-matched men) were studied. Participants were excluded for: history of myocardial infarction, heart failure, cardiovascular diseases, diabetes, chronic diseases, and current tobacco use. Cutaneous microvascular function was measured as previously described^[30,31]^. Descriptive data for participants is presented in Supplementary Table 2.

Females were studied during the early follicular phase of the natural menstrual cycle or in the placebo pill phase of oral contraceptive pill use to minimize the influence of female sex hormones. Participants arrived at the laboratory after an overnight fast and abstained from alcohol/caffeine for 12 h and vigorous exercise for 24 h. Height, weight, and resting blood pressure were measured. Two microdialysis fibers (CMA 31; Harvard Apparatus, Holliston, MA) were inserted in the ventral side of the non-dominant forearm of each participant, as previously described^[30–33]^. A 25-gauge needle was inserted into the dermis after a 10-min application of ice to the skin surface to provide short-term local anesthesia. The MD fibers were then threaded through the lumen of the needle, which was removed once the semi-permeable section of the fiber was in place. The MD fibers were taped to the skin, and Ringer’s solution was perfused through all sites for at least 60 min or until the local hyperemia associated with fiber insertion subsided.

Skin blood flow was measured as cutaneous red blood cell (RBC) flux from 1.5 mm^2^ of skin with a multifiber laser Doppler probe placed in a local heater (MoorLAB, Temperature Monitor SH02, Moor Instruments, Devon, UK). Brachial blood pressure was measured every 10 min on the contralateral arm by an automated oscillometric sphygmomanometer (GE Healthcare, Dinamap ProCare 400 Vital Signs Monitor, Chicago, IL, USA). Following the hyperemia resolution period, one site was randomly assigned to receive the AT2R antagonist PD123319 (1 μM, Tocris) and the other site received Ringer’s solution as a control. Following 1 h of pre-treatment with PD123319 or Ringer’s solution, both sites received the same incremental concentrations of AngII from 10^−9^ to 10^−4^ M to elicit vasoconstriction^[16]^.

RBC flux data were collected at 40 Hz using the PL3516 PowerLab data acquisition system and LabChart software (AD Instruments, Colorado Springs, CO). Cutaneous vascular conductance (CVC) was calculated as RBC flux divided by mean arterial pressure and standardized to site-specific baseline (%CVC_baseline_).

### Human cell culture studies and immunoblotting

De-identified human aortic tissue was obtained post-mortem from the NIH-supported National Disease Research Interchange, and hence, the medical ethics committee of participating center (Tufts Medical Center) deemed this research to be exempt from human subjects’ research requirements. Human aortic smooth muscle cells (HAoSMC) and endothelial cells (HAoEC) from premenopausal women and age-matched men were compared. For HAoEC isolation, aorta tissues were enzymatically digested by dispase (Gibco) to create single EC suspensions. Pure ECs were isolated using magnetic beads conjugate EC-specific antibody (Invitrogen). HAoECs were cultured in endothelial cell medium (Cell Biologics) and grown to ~90% confluency before collection. HAoSMC were isolated by the explant method, as previously described^[18]^, and used only to passage 8.

The cell lysates in protein sample buffer were incubated for 5 min at 95 °C, run on a 10% SDS PAGE gel, transferred to PVDF Transfer Membrane (Millipore), blocked with 5% non-fat milk, and probed with appropriate primary antibodies (AT2R and AT1R, Abcam; GAPDH, Cell Signaling). Secondary antibodies used were anti-mouse and anti-rabbit horseradish peroxidase secondary antibody (Cell Signaling). Blots were imaged using ECL reagent (Fisher).

### Statistical analysis

Group differences were assessed using one-way analysis of variance (ANOVA), two-way ANOVA, repeated-measures two-way ANOVA, or Student’s *t*-test as indicated in each figure legend. Tukey post hoc testing was used for all post hoc pairwise comparisons. Effect size (Cohen’s *d*) and 95% confidence interval (CI: lower limit, upper limit) are displayed as necessary for data transparency. The level of significance was set at α = 0.05 for all statistical tests. All data are presented as mean ± standard error of the mean (SEM).

## RESULTS

### Angiotensin II-induced vasoconstriction increases later in life in female compared with male mice

**Figure 1.**
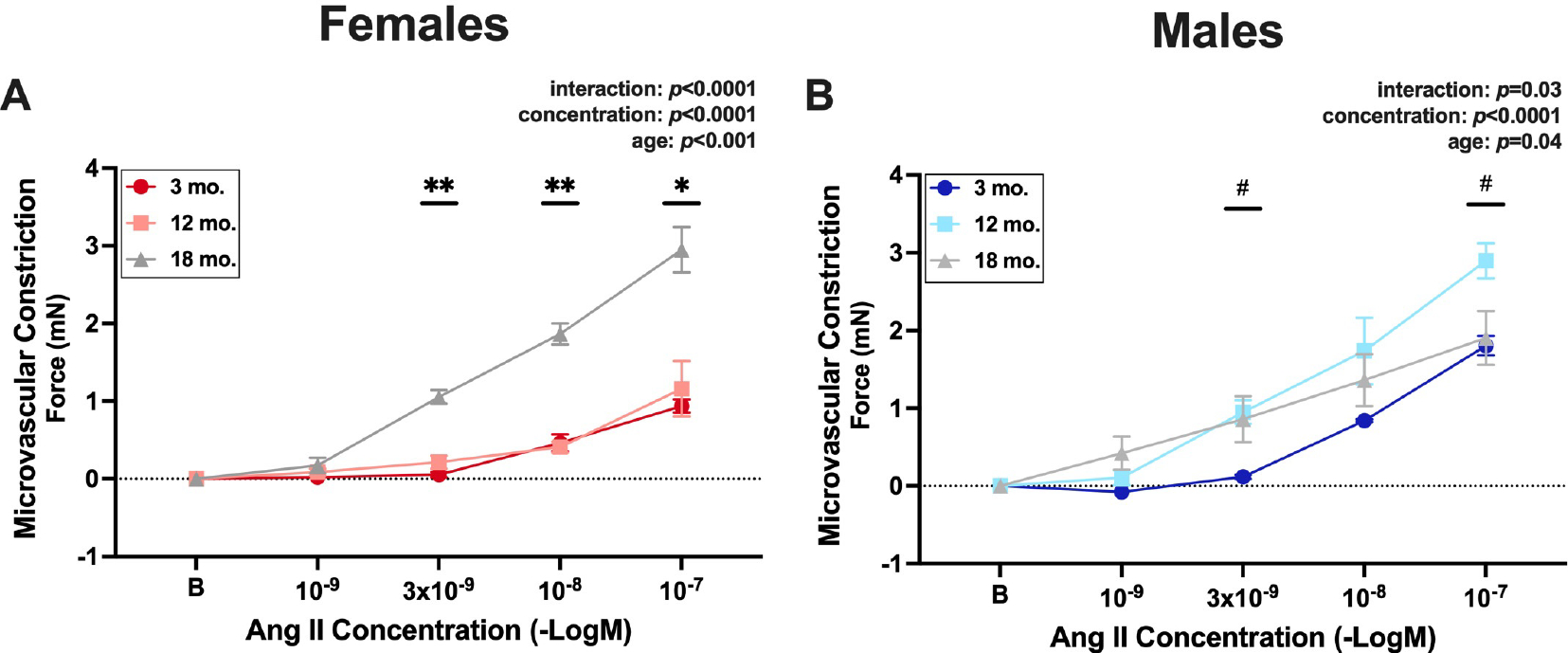
Angiotensin II-induced vasoconstriction increases later in life in female compared with male mice. Second- and third-order mesenteric resistance arteries were isolated from 3-, 12-, and 18-month-old C57/Bl6 mice. Four separate segments from each mouse were hung in a wire myograph and administered a different concentration of angiotensin II (1 × 10^−9^ to 1 × 10^−7^ M). Microvascular constriction was measured in vessels from (A) females and (B) males in response to angiotensin II concentrations by wire myography. Data are expressed as raw force in milliNewtons (mN) from baseline. *N* = 3–4/group (all female groups and 3-month-old males = 3/group; 12- and 18-month-old males = 4/group). The main effects of concentration and age and the interaction effect of these two factors were assessed via repeated measures two-way ANOVA with Tukey post hoc testing where appropriate. Data are means ± SEM, *P < 0.05, 18-month-old females *vs*. all other female groups. **P < 0.01, 18-month-old females *vs*. all other female groups. ^#^*P* < 0.05, 3-month-old males *vs*. 12-month-old males.

To examine sex-specific, aging-related changes in microvascular AngII-induced constriction, MRA were isolated from 3, 12, and 18-month-old female and male mice and constriction to AngII was determined. The response to AngII is a composite measure of constriction driven by the AT1R and dilation due to AT2R activity, with AT1R function dominating and resulting in an overall constrictive response. In female mice, AngII-induced constriction was greater in 18-month-old compared with both 3- and 12-month-old mice, with no difference in vasoconstriction at any concentration between 3- and 12-month-old mice [Figure 1A]. In male mice, AngII-induced constriction increased from 3 to 12 months of age, but vasoconstriction declined by 18 months of age, such that it was no longer significantly different from 3-month-old mice [Figure 1B].

### AT2R-mediated vasodilation declines with age in female mice only

**Figure 2.**
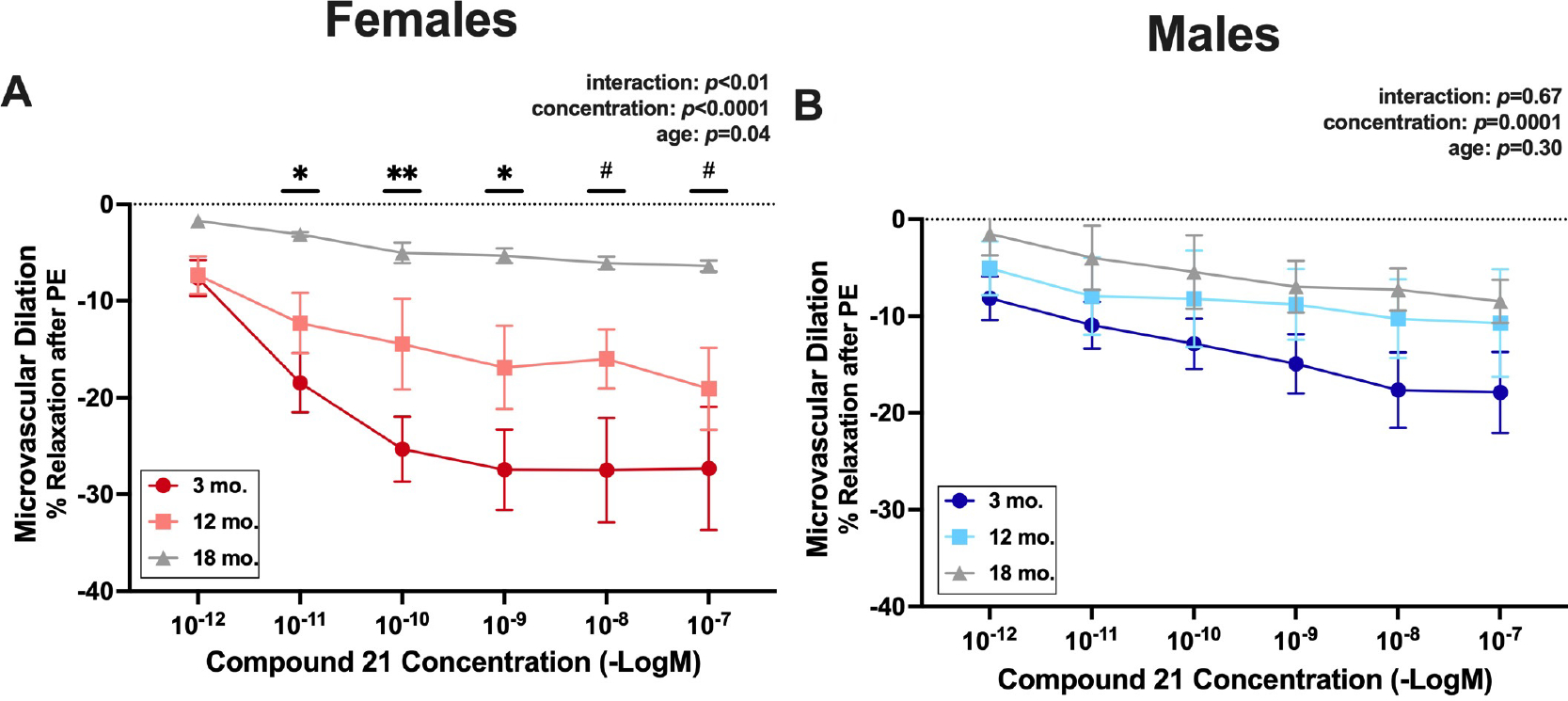
AT2R-mediated vasodilation declines with age in female mice only. Second- and third-order mesenteric resistance arteries were isolated from 3-, 12-, and 18-month-old C57/Bl6 (A) female and (B) male mice. Vessels were pre-treated with losartan for 30 min to block angiotensin II type 1 receptors. Following preconstriction to phenylephrine (PE), vasorelaxation in response to Compound 21 (AT2R agonist, 1 × 10^−12^ to 1 × 10^−7^ M) was determined by wire myography. Data are expressed as percent relaxation from PE preconsriction. *N* = 3–7/group (18-month-old males and females = 3/group; 3-month-old females and males and 12-month-old males = 4/group; 12-month-old females = 7/group). The main effects of concentration and age and the interaction effect of these two factors were assessed via repeated measures two-way ANOVA with Tukey post hoc testing where appropriate. Data are means ± SEM, *P < 0.05, 3-month-old females *vs*. 18-month-old females; **P < 0.01, 3-month-old females *vs*. 18-month-old females; ^#^*P* < 0.05, 12-month-old females *vs*. 18-month-old females.

To determine if differences in AT2R activity contribute to the sex differences in microvascular function, MRA were pre-treated with losartan to block AT1R, preconstricted with PE, and then administered increasing concentrations of AT2R agonist C21. There were no statistical differences in the preconstriction response to PE between compared groups (young females: 3.1 mN ± 0.4, aged females: 3.2 mN ± 0.3, young males: 2.9 mN ± 0.2, aged males 3.2 mN ± 0.3). In female mice [Figure 2A], there was no difference in AT2R-mediated dilation at any concentration between 3 and 12-month-old mice. Compared with 18-month-old female mice, AT2R-mediated dilation was greater in 3-month-old mice at lower concentrations (10^−11^-10^−9^ M) and greater in 12-month-old mice at higher concentrations (10^−8^-10^−7^ M). There was no effect of age on AT2R-mediated dilation in male mice [Figure 2B].

### Mesenteric resistance artery AT2R mRNA declines with age in female mice only

**Figure 3.**
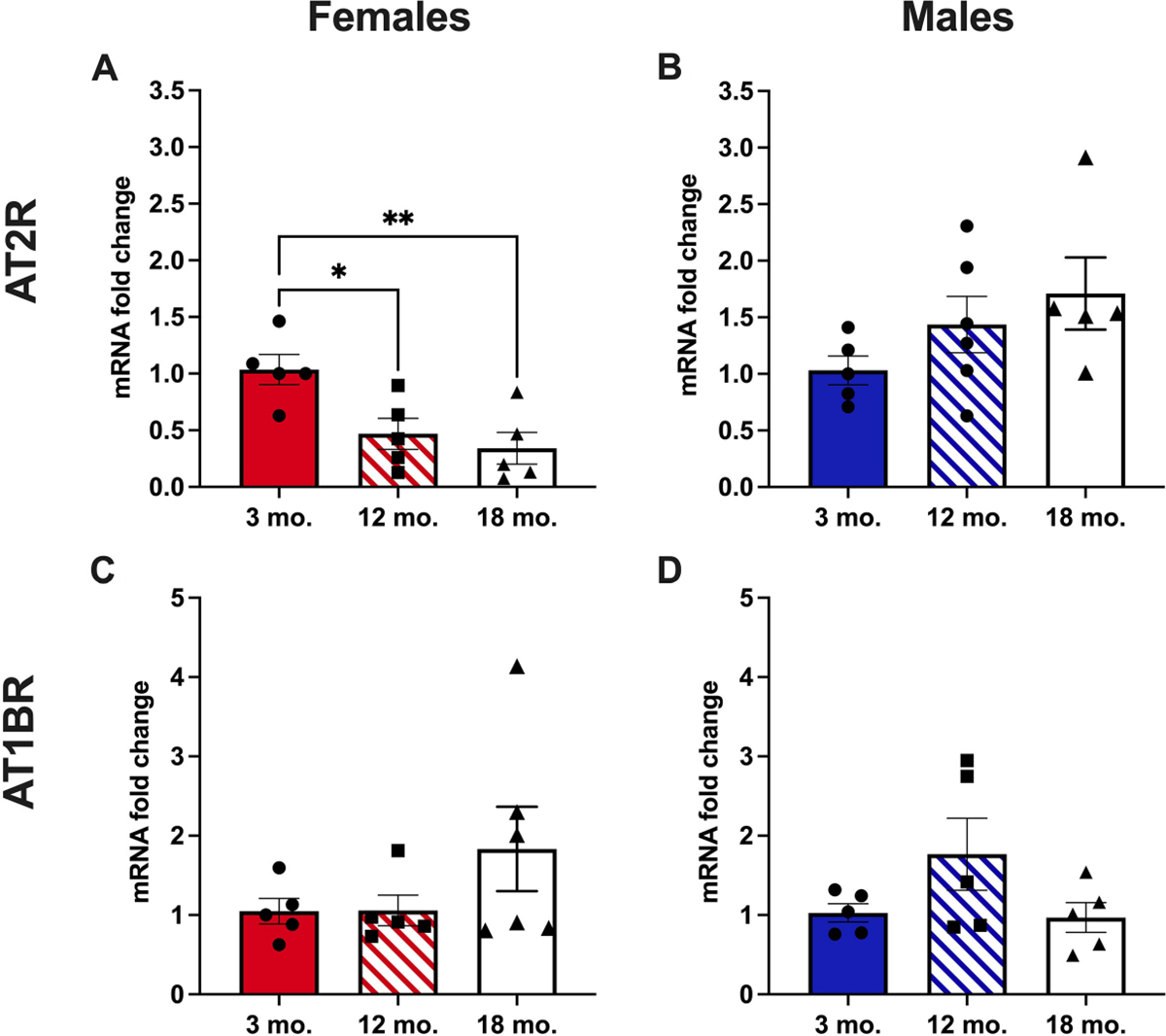
Mesenteric resistance artery AT2R mRNA declines with age in female mice only. Total RNA was extracted from mesenteric resistance arteries, and qRT/PCR was performed to quantify angiotensin II type 2 [AT2R, (A and B)] and type 1b [AT1BR, (C and D)] receptor mRNA expression. *N* = 5–6/group. Differences were assessed via one-way ANOVA with Tukey post hoc testing where appropriate. Data are means ± SEM, *P < 0.05; **P < 0.01.

Next, we examined whether the age-dependent changes in AT2R activity may be mediated by changes in MRA AT2R expression. AngII receptor mRNA expression was quantified in mouse MRA [Figure 3]. In female mice, AT2R mRNA decreased in 12- and 18-month-old mice *vs*. 3-month-old mice [Figure 3A]. There was no difference in AT2R expression with age in MRA from male mice [Figure 3B]. We observed no difference in AT1RB mRNA expression with aging in female [Figure 3C] or male [Figure 3D] mice.

### Angiotensin II-induced vasoconstriction is attenuated in young female compared with male mice, and this difference is abolished with AT2R inhibition

**Figure 4.**
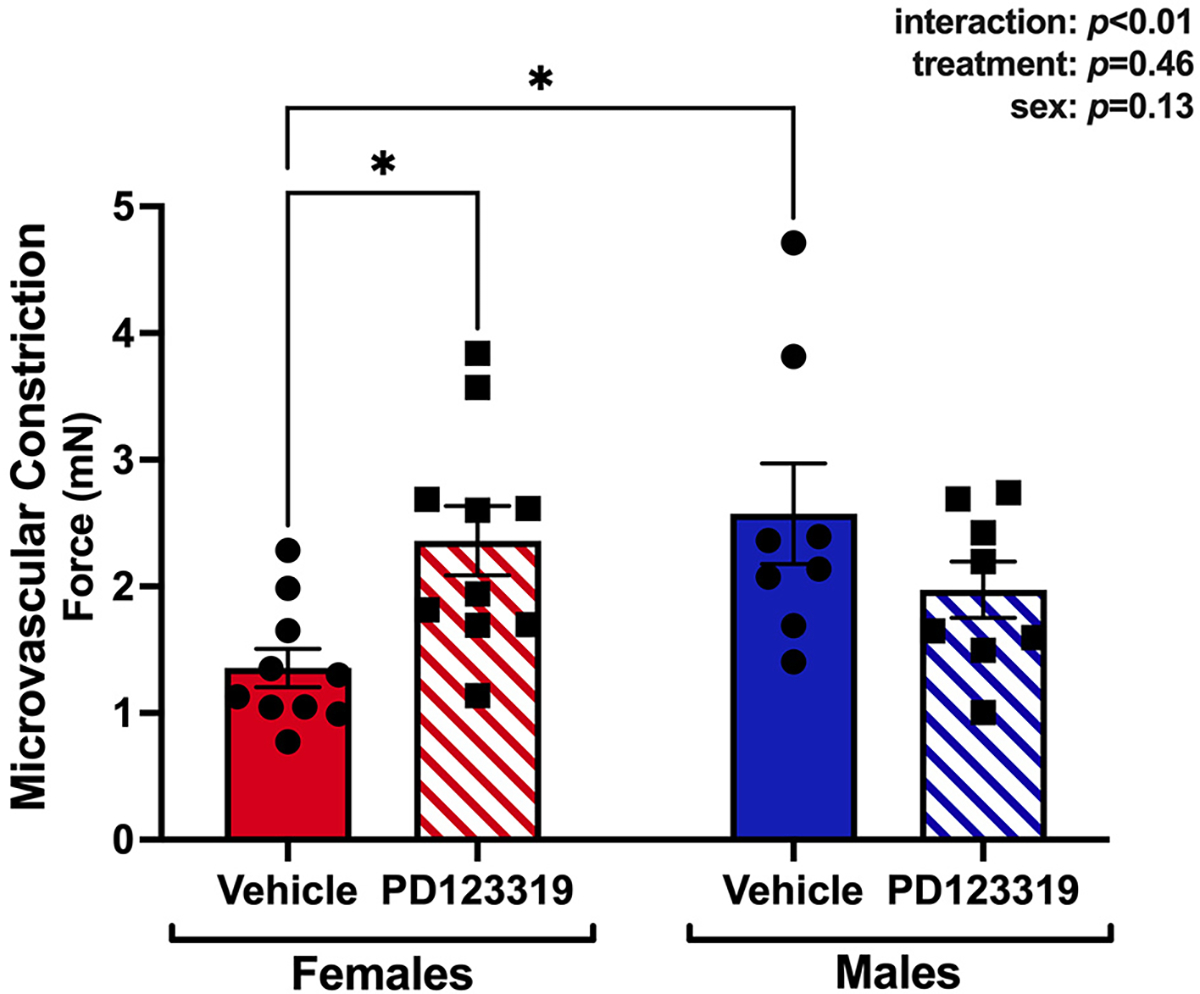
Angiotensin II-induced vasoconstriction is attenuated in young female compared with male mice, and this difference is abolished with AT2R inhibition. Second- and third-order mesenteric resistance arteries were isolated from 3-month-old C57/Bl6 female and male mice. Vessels were pre-treated with vehicle (circles) or 10 μM PD123319 (squares), an AT2R antagonist. Vessel constriction in response to AngII (1 × 10^−7^ M) was determined by wire myography. Data are expressed as raw force in milliNewtons (mN). *N* = 8–10/group. The main effects of treatment and sex and the interaction effect of these two factors were assessed via two-way ANOVA with Tukey post hoc testing where appropriate. Data are means ± SEM, *P < 0.05.

To determine if AT2R activity mediates sex differences in AngII-induced constriction, wire myography studies were performed to quantify AngII-induced constriction (AngII dose 10^−7^ M) with and without pretreatment with the AT2R inhibitor, PD123319, in MRA from young female and male mice. We chose a 10^−7^ M dose of AngII, because it was the highest dose used in the studies from Figure 1 and where we observed the most robust differences between ages. In vehicle pre-treated vessels, AngII-induced constriction was attenuated in young females *vs*. males. AT2R inhibition enhanced AngII constriction in females only. There was no effect of AT2R inhibition on AngII-induced constriction in males [Figure 4].

### Angiotensin II-induced vasoconstriction is attenuated in premenopausal women compared with men, and this difference is abolished with AT2R inhibition

**Figure 5.**
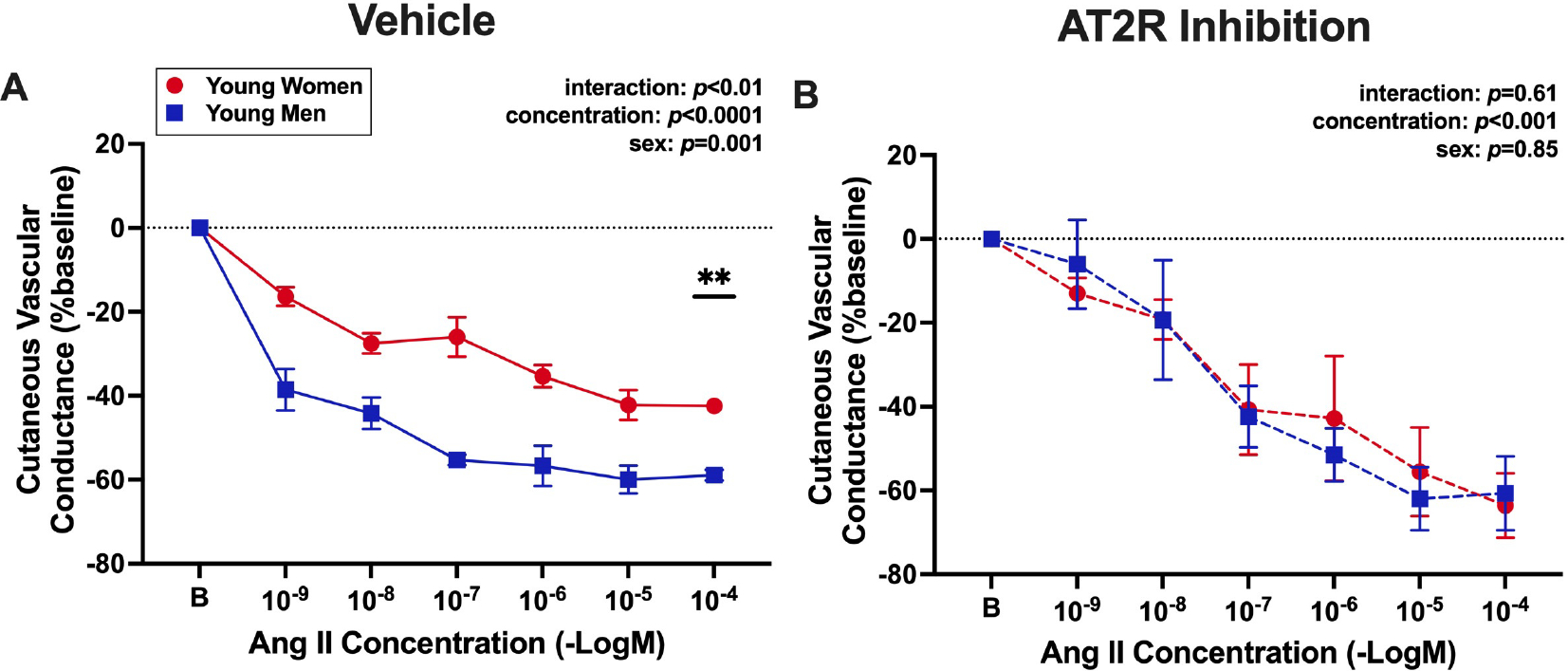
Angiotensin II-induced vasoconstriction is attenuated in premenopausal women compared with men, and this difference is abolished with AT2R inhibition. Forearm cutaneous vascular conductance was assessed to measure microvascular constriction in response to increasing concentrations of AngII (1 × 10^−9^ to 1 × 10^−4^ M) in young, premenopausal women (*N* = 3, red circles, average age: 28 ± 2 years) and young, age-matched men (*N* = 4, blue squares, average age: 30 ± 3 years). Local skin sites were pre-treated with (A) lactated Ringer’s (vehicle control) or (B) an AT2R antagonist (1 μM PD123319). Cutaneous vascular conductance (red blood cell flux/mean arterial pressure) is expressed as a percentage of baseline. The decline in cutaneous vascular conductance in response to AngII corresponds to the degree of vasoconstriction of the cutaneous microvasculature. The main effects of concentration and sex and the interaction effect of these two factors were assessed via repeated measures two-way ANOVA with Tukey post hoc testing where appropriate. Data are means ± SEM, **P < 0.01.

To translate murine findings to humans, cutaneous microvascular constriction to AngII in the presence and absence of AT2R antagonist, PD123319, was assessed in healthy women and men. For the presentation of data in Figure 5, the decline in cutaneous vascular conductance in response to AngII corresponds to the degree of vasoconstriction of the cutaneous microvasculature. There was a significant effect of sex at vehicle sites, where women constricted less to AngII compared with men [Figure 5A]. There was also a significant interaction effect of sex and concentration at vehicle sites [Figure 5A], such that vasoconstriction at 10^−4^ M AngII was reduced in women (−42 ± 1 %CVC_baseline_) compared with men (−59 ± 1 %CVC_baseline_, *P* < 0.01). At sites pre-treated with PD123319, there was no difference in AngII-induced constriction between women and men at any concentration [Figure 5B]. In women only, there was a large effect size comparing AngII vasoconstriction at 10^−4^ M between vehicle and AT2R-inhibited sites (*d* = 3.9; 95%CI: −53, 11 %CVC_baseline_), supporting a physiological role and protective effect of AT2R activity in the microvasculature of premenopausal women.

### AT2R expression in human aortic smooth muscle cells is greater in premenopausal women *vs*. men

**Figure 6.**
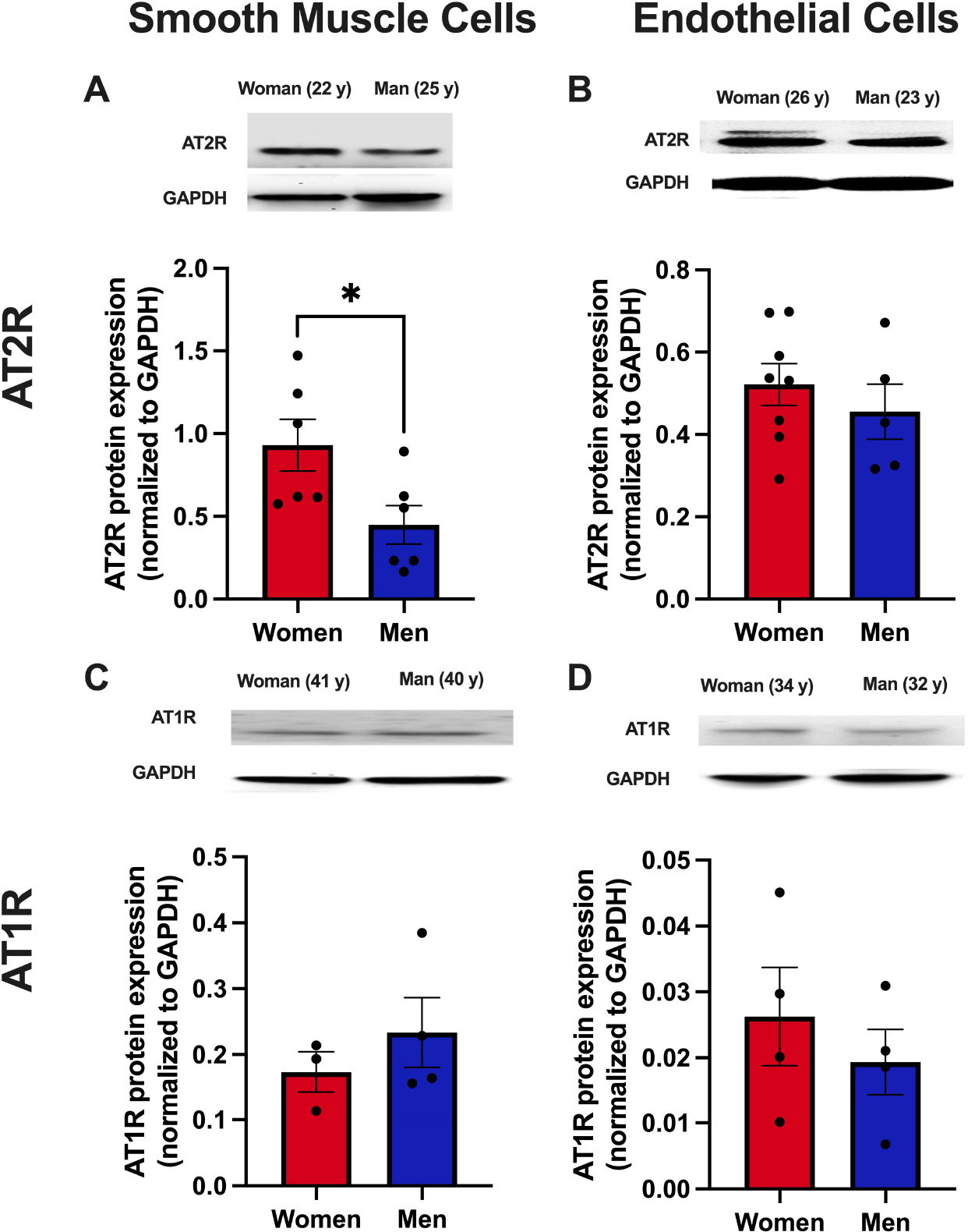
AT2R expression in human aortic smooth muscle cells is greater in premenopausal women *vs*. men. The expression of angiotensin II type 2 [AT2R, (A and B)] and type 1 [AT1R, (C and D)] was quantified in primary human aortic smooth muscle cells (A and C) and endothelial cells (B and D). Samples are from adult women (average age: 37 ± 1 years) and men (average age: 34 ± 2 years) donors. Representative immunoblots and quantification are shown. *N* = 3–8 samples per group. Differences were assessed via the student’s *t*-test. Data are means ± SEM for protein expression normalized to GAPDH, *P < 0.05.

To elucidate whether there are sex differences in vascular cell expression of AngII receptors, protein expression of AT2R and AT1R was determined in primary HAoSMC and HAoEC from young women (37 ± 1 years) and men (34 ± 2 years). There was greater protein expression of AT2R in HAoSMC from premenopausal women compared with age-matched men [Figure 6A]. There were no sex differences in AT2R expression in HAoEC [Figure 6B] or in AT1R expression in HAoSMC [Figure 6C] or HAoEC [Figure 6D].

## DISCUSSION

The novel findings from the present study are that: (1) in female mice, there is a concurrent loss of microvascular AT2R-mediated dilation and an increase in AngII-induced constriction with advancing age; (2) microvascular AngII-induced constriction increases earlier in males with no aging-associated change in AT2R-mediated dilation; (3) the decline of AT2R-mediated dilation with aging in female mice corresponds with a decrease in microvascular AT2R mRNA expression; (4) young female mice have attenuated microvascular AngII-induced constriction compared with young male mice, and this sex difference is driven by enhanced AT2R function; and (5) clinical data from premenopausal women and age-matched men support the sex difference in microvascular AT2R function seen in mice, such that young women have attenuated AngII-induced constriction compared with men, which is abolished with AT2R inhibition, indicating enhanced AT2R function in young women. This is associated with greater AT2R protein expression in HAoSMC from premenopausal women. Overall, these findings demonstrate that young females have enhanced microvascular AT2R expression and function, and that female microvascular aging is driven by both a loss of AT2R-mediated dilation and an increase in AngII-induced vasoconstriction, while male microvascular aging is primarily driven by an increase in AngII-induced vasoconstriction with no alterations in AT2R function.

The prevalence of cardiovascular disease increases with aging in both men and women, but this occurs in a sexually dimorphic pattern^[1]^. In general, premenopausal women are protected from cardiovascular disease and develop CVD later in life compared with men^[1]^. However, the mechanisms that mediate the sex-specific time course of CVD progression are not well understood. There are validated sex differences in the RAAS and known changes in levels of RAAS components (including AngII and its receptors) with aging^[4,34]^. However, there has been limited investigation of sex differences in vascular RAAS alterations with aging, specifically in the microcirculation. Prior studies have suggested that sex differences in responses to AngII may be mediated by differences in AT1R expression and activity or AngII synthesis, which can be regulated by sex hormone exposure^[21,35]^. Findings from the current study identify differences in AT2R function and expression as a sex-specific mechanism of microvascular aging.

Previous work shows that the AT2R contributes to vasodilation under healthy conditions^[7–11]^. There is evidence in various vascular beds that AT2R stimulation yields vasodilation via bradykinin, nitric oxide (NO), cyclic guanosine monophosphate, and cytochrome P-450-dependent (but NO-independent) pathways^[36]^. More specifically, C21-mediated activation of AT2R induces NO release through PKA/phosphorylated endothelial-derived NO synthase (p-eNOS) and AKT/p-eNOS signaling pathways^[37]^. Previous studies have identified a vasodilatory role of AT2R in the microvasculature of normotensive male rats, but this is reversed to a vasoconstrictive role in the setting of aging^[38]^ and hypertension^[10,12]^. Importantly, AT2R function is restored to a vasodilatory response when blood pressure is normalized in the aging and hypertensive models^[10,12,38]^. The current findings are consistent with previous reports, as our results show that males exhibit only modest AT2R-mediated dilation at any age. One difference is that our study directly measured AT2R-mediated dilation with C21, whereas the previous studies assessed the vascular response to AngII in the presence and absence of AT2R inhibition, which may contribute to slightly differing results in young males between the current study and previous findings. The alterations we observed in AT2R function in females coincided with a reduction in microvascular AT2R mRNA expression in females but not males. The present study also provides evidence that AT2R represses microvascular AngII-induced constriction in young female but not male mice. These findings support that microvascular AT2R function is enhanced and protective in young females but not males. Clinical data demonstrate that women are more likely to have non-obstructive coronary artery disease attributable to coronary microvascular dysfunction^[39]^, for which there are no current sex-specific treatment options. The current findings suggest that restoration of AT2R expression and function may be an effective sex-specific treatment in the management and prevention of aging-associated cardiovascular disease in women. It is also plausible that AT2R agonist therapy may be beneficial in males, but future studies are required to determine this.

There is strong evidence that sex differences in AT2R function contribute to blood pressure responses in the setting of RAAS activation. Multiple studies have shown that young female rodents have a reduced pressor response to AngII compared with males^[11,20–22]^. This has also been shown in response to acute AngII infusion in young, healthy women compared with men^[40]^. Young female mice exhibit a decrease in mean arterial pressure in response to low-dose AngII, which can be reversed by AT2R antagonism, while males have no response^[11,41]^. This AT2R-mediated reduction in arterial pressure is estrogen-dependent in females^[42]^. Similarly, aged, reproductively senescent females also demonstrate an AT2R-mediated reduction in AngII-induced increases in blood pressure when supplemented with estrogen^[43]^. Cerebral AT2R is also protective against the development of mineralocorticoid receptor- plus salt-induced hypertension in female, but not male, rats^[44]^. In the renal system, AT2R in female rodents maintains autoregulation of renal blood flow and glomerular filtration rate at low renal perfusion pressures^[45]^, counteracts renal pressor responses to AngII^[45]^, and mediates the pressure-natriuresis relationship, such that young females excrete the same amount of sodium as young males but at a lower arterial pressure^[46]^. Interestingly, the AT2R-dependent effect on the pressure-natriuresis relationship is lost with aging in female rodents, which coincides with a reduction in the ratio of AT2R to AT1R mRNA in the kidney with aging^[46]^. Findings from the present study further our understanding of how sex differences in AT2R function may contribute to hypertension and cardiovascular disease, adding that the loss of enhanced microvascular AT2R activity in females may contribute to the aging-associated increase in hypertension and cardiovascular diseases in aging women.

Obesity and pre-diabetes are also risk factors for cardiovascular disease and increase with aging. Obesity is also more common in women^[1]^, and when associated with pre-diabetes, obesity mitigates the protection from CVD seen in premenopausal women^[47]^. The development of obesity with pre-diabetes in rats reduces cardiac AT2R expression in females but not males^[48]^. Further, increasing AT2R expression in the heart can mitigate cardiac damage induced by obesity and pre-diabetes in male and female rats^[49,50]^. Future studies are warranted to determine whether exposure to obesity accelerates the aging-related decline in microvascular AT2R function as a mechanism for promoting cardiovascular disease risk in women.

There are substantial available data demonstrating a role for sex hormones and sex hormone receptors in regulating AT2R expression. Overall, expression of AT2R is greater in females compared with males in many tissues, including the kidney, heart, vasculature, adrenal glands, and the central and peripheral nervous systems^[9,11,48]^. Several mechanisms have been implicated in determining this sex difference^[9]^. First, the gene encoding for AT2R resides on the X chromosome^[24]^; hence, the dosage effect of the two X chromosomes may contribute to greater expression in females. There are also estrogen-responsive elements in the promoter region of the AT2R gene, and estrogen upregulates the expression of AT2R in many tissues^[9,26,51,52]^. Conversely, testosterone downregulates AT2R expression in the aorta^[25]^, which may contribute to lower expression of AT2R in males. Upregulation of AT2R expression by estrogen is dependent on estrogen receptor β in uterine arteries from pregnant rats^[26]^ and downregulation of AT2R by testosterone is androgen receptor-dependent in the aorta of female rats^[25]^. Protection from AngII-induced increases in blood pressure in female rodents is also estrogen receptor α-dependent^[21]^. The average age of cessation of normal estrous cycles in female C57/Bl6 mice occurs between 11 and 15 months of age^[27]^. Therefore, a reduction in circulating estrogens may contribute to the results in the present study, though this cannot be confirmed with the present data, as we did not measure circulating estrogens in our female mice. Future studies on the role of sex hormones and sex hormone receptors on AT2R expression and function in the microvasculature during aging are warranted.

Our clinical study adds to the growing literature supporting the role of AT2R in microvascular function in women. As we show in young female mice, premenopausal women have attenuated microvascular constriction to AngII compared with age-matched men, and this difference is abolished with AT2R inhibition. Other clinical studies have recently demonstrated both sex- and aging-related differences in AT2R function in human cutaneous microvascular responses. Lang and Krajek^[16]^ found that AT2R activity attenuates microvascular AngII-induced constriction in mixed-sex cohorts of young adults but not older adults. More recently, Schwartz *et al.* found that AT2R-mediated dilation in response to C21 is greater in young women compared with young men^[17]^. An earlier study by Stewart *et al*. showed that AngII increased microvascular vasodilation during concurrent AT1R inhibition and NO synthase inhibition, but that additional inhibition of AT2R (with PD123319) had no effect^[53]^. This study was completed in a young, mixed-sex cohort (5 men, 3 women)^[53]^, and hence, it remains unclear how sex differences may have affected this outcome. Overall, our results identify a novel mechanism driving the sex difference in the cutaneous microvascular response to AngII, with attenuated vasoconstriction in premenopausal women that is attributable to enhanced AT2R activity.

Our data also show greater AT2R protein expression in HAoSMC from premenopausal women compared with men, but no differences in AT2R expression in HAoEC or AT1R expression in either cell type. This suggests a greater AT2R to AT1R ratio in smooth muscle cells from premenopausal women compared with men, which may contribute to sex differences in AngII effects on vascular function outcomes in humans. These data also suggest that AT2R in SMC, but not EC, may be a potential driver of cardiovascular protection in young females. However, previous literature also suggests that AT2R may have varying effects by vascular bed, as estrogen supplementation in aged, reproductively-senescent female mice showed AT2R-dependent attenuation in AngII-induced increases in blood pressure but had no effect on aortic vasodilator function^[43]^. It is important to note that the present studies assessing human protein expression of AngII receptors were completed with aortic vascular cells, and further studies are needed to confirm these findings in human microvascular SMC and EC, which are less readily available.

### Limitations

This study has several limitations that should be considered when interpreting the results. First, we did not measure blood pressure in the murine aging model; however, previous studies have shown that young male mice have modestly higher basal blood pressure than young female mice^[54]^ and that male mice develop a modest increase in blood pressure by 7 months of age^[55]^. Potential aging-associated alterations in blood pressure in female mice are still unknown and warrant future studies. Third, we did not measure the protein expression of AngII receptors in mice. This is largely due to the lack of reliable antibodies for AT1R in mice. In addition, our study design did not address how endothelial function may influence AT2R-mediated vasodilation. Future studies are required to dissect the role of endothelial function in age and sex differences in AT2R-mediated vasodilation. Next, due to limited recruitment capacity, we were not able to measure human microvascular function in older adults; thus, further investigation of sex differences in microvascular AngII receptor activity in human aging is necessary. Additionally, we measured AngII receptor expression in human aortic cells, which may differ from what occurs at the microvascular level in humans. Nonetheless, we did detect sex differences in aortic SMC AT2R expression, suggesting that aortic AT2R may also be relevant in female vascular health. Lastly, we recognize that we have low sample sizes in some of our mouse mesenteric function data. Although our data are significant, future studies should confirm with larger sample sizes.

### Perspectives

Overall, the present study identifies enhanced microvascular AT2R activity in young female compared with male mice and demonstrates a decline in microvascular AT2R expression and AT2R-mediated vasodilation as a novel mechanism of female vascular aging. The clinical data provide novel translational evidence of sex differences in AT2R function in the human microvasculature. These results nominate the AT2R as a potential sex-specific therapeutic target for microvascular dysfunction and the consequential development of cardiovascular disease in aging women. As AT2R agonists have already been successfully used in clinical trials in humans, these findings have the potential to be rapidly translated. Future investigation is essential to determine the precise molecular mechanisms regulating microvascular AT2R function and how this differs by sex and age to contribute to the differential manifestation of aging-associated cardiovascular diseases in women and men.

## Supplementary Material

Supplementary Material

## Data Availability

Not applicable.
