# Supplementary material for: Microvascular angiotensin II type 2 receptor function is enhanced in young females and declines in a model of murine aging
Source: J Cardiovasc Aging. Author manuscript; Available in PMC 2025 Apr 25. (PMC12030185; doi:10.20517/jca.2024.09)
Supplement: Supplementary Material [file NIHMS2017527-supplement-Supplementary_Material.pdf]

Supplemental Material

Microvascular angiotensin II type 2 receptor function is enhanced in young females and declines in a model of murine aging

Casey G. Turner[1], Karla de Oliveira[1], Qing Lu[1], Ayan R. Patel[2], Lakshmi Pulakat[1], Iris Z. Jaffe[1], Jennifer J. DuPont[1]

[1]Molecular Cardiology Research Institute, Tufts Medical Center, Boston, MA 02111, USA.
[2]Division of Cardiology, Department of Medicine, Tufts Medical Center, Boston, MA 02111, USA.

Correspondence to: Dr. Jennifer J. DuPont, Molecular Cardiology Research Institute, Tufts Medical Center, 800 Washington Street, Box 80, Boston, MA 02111, USA. E-mail: Jennifer.dupont@tuftsmedicine.org

Table S1. Gene names, abbreviations, and primer sequences.

Gene Name	Gene Symbol	Primer Sequence (5' – 3')
Angiotensin II type 2 receptor	AT2R	
Forward		ATGCTTGTATTATGGCTTTCCCA
Reverse		TTGGTCACGGGTAATTCTGTTC
Angiotensin II type 1 B receptor	Agtr1B	
Forward		TGGCTTGGCTAGTTTGCCG
Reverse		ACCCAGTCCAATGGGGAGT
Beta-2-microtubulin	B2m	
Forward		GCTATCCAGAAAACCCCTCAA
Reverse		CATGTCTCGATCCCAGTAGACGGT

Table S2. Human participant characteristic information.

	Women (N=3)	Men (N=4)
Age (y)	28 ± 2	30 ± 3
Body mass index (kg/m^2)	23 ± 1	23 ± 1
Mean arterial pressure (mmHg)	78 ± 4	87 ± 3
